# Prognostic Significance of STING Expression in Extramammary Paget’s Disease

**DOI:** 10.3390/cancers18081314

**Published:** 2026-04-21

**Authors:** Yoko Amagata, Natsuko Saito-Sasaki, Yu Sawada

**Affiliations:** Department of Dermatology, University of Occupational and Environmental Health, 1-1, Iseigaoka, Yahatanishi-Ku, Kitakyushu 807-8555, Fukuoka, Japan

**Keywords:** extramammary Paget’s disease, STING, prognosis

## Abstract

**Background/Objectives**: Extramammary Paget’s disease (EMPD) is a rare cutaneous malignancy with a highly variable clinical behavior. While early-stage disease is often indolent, progression to dermal invasion and lymph node metastasis is associated with a poor prognosis. Reliable biomarkers that reflect tumor aggressiveness and predict clinical outcomes are lacking. The stimulator of interferon genes (STING) pathway plays a central role in innate immune signaling and antitumor immunity; however, its clinical significance in EMPD remains unclear. **Methods**: We retrospectively analyzed STING expression using immunohistochemistry in formalin-fixed, paraffin-embedded tumor specimens obtained from 63 patients with EMPD. Associations between STING expression and clinicopathological features were evaluated. Overall survival was analyzed using Kaplan–Meier methods, and prognostic factors were assessed using univariate and multivariate Cox proportional hazards regression analyses. **Results**: STING expression was positive in 26 (41%) and negative in 37 (59%) patients. Loss of STING expression was significantly associated with dermal invasion and lymph node swelling. Kaplan–Meier analysis demonstrated a significantly poorer overall survival in patients with STING negativity than in those with STING positivity. In univariate analysis, lymph node swelling, dermal invasion, and STING expression were significantly associated with survival. Multivariate Cox regression analysis identified lymph node swelling as an independent adverse prognostic factor, whereas STING expression was an independent favorable prognostic factor. Loss of STING expression is closely associated with aggressive clinicopathological features and independently predicts poor prognosis in EMPD. **Conclusions**: STING expression may serve as a useful prognostic biomarker and provides insight into the immunobiology of EMPD.

## Simple Summary

Extramammary Paget’s disease (EMPD) is a rare skin cancer with highly variable clinical outcomes. While some patients show slow disease progression, others develop invasive disease with poor prognosis. Currently, reliable biomarkers to predict disease aggressiveness are limited. In this study, we investigated the expression of STING, a key molecule involved in immune responses, in tumor tissues from patients with EMPD. We found that loss of STING expression was associated with more aggressive disease features, including dermal invasion and lymph node involvement. Importantly, patients with negative STING expression showed significantly worse survival compared to those with positive expression. These findings suggest that STING expression may serve as a useful biomarker for predicting prognosis and may provide new insights into the biological behavior of EMPD.

## 1. Introduction

Extramammary Paget’s disease (EMPD) is a rare cutaneous malignancy that primarily affects older adults and commonly develops in apocrine gland-bearing skin [1,2]. Although EMPD is often indolent at an early stage, disease progression characterized by dermal invasion and lymph node metastasis is associated with a markedly poor prognosis [3]. Once regional or distant metastasis occurs, therapeutic options become limited and clinical outcomes remain unfavorable [4]. Therefore, identification of reliable biomarkers that reflect tumor aggressiveness and predict patient prognosis is a critical unmet need in the management of EMPD.

The current prognostic assessment of EMPD relies primarily on clinicopathological parameters, such as dermal invasion and lymph node involvement [3,4]. While these factors are clinically useful, they may not fully reflect the biological heterogeneity of EMPD. Moreover, conventional histopathological features do not adequately reflect tumor–host immune interactions, which are key determinants of cancer progression and patient survival.

The stimulator of interferon genes (STING) pathway is the central component of innate immune sensing and plays a pivotal role in antitumor immunity [5,6]. Activation of STING signaling induces type I interferon production and promotes dendritic cell activation, cytotoxic T-cell priming, and immune-mediated tumor control [7,8]. Dysregulation of STING signaling has been implicated in immune evasion and tumor progression in various malignancies. However, the biological and prognostic significance of STING signaling is highly context- and tumor-type-dependent [9,10]. Notably, in hematological malignancies including lymphomas, a reduced STING expression in tumor cells has been associated with an aggressive disease behavior and unfavorable prognosis [11]. However, the clinical relevance of STING expression in EMPD has not been systematically investigated.

Given the growing recognition of the importance of the tumor immune microenvironment in cutaneous malignancies, we hypothesized that STING expression may reflect disease progression and clinical outcomes in EMPD. In this study, we examined STING expression using immunohistochemistry in a cohort of patients with EMPD and analyzed its association with clinicopathological features and patient survival. Furthermore, we evaluated the prognostic significance of STING expression using univariate and multivariate Cox regression analyses to determine whether STING expression serves as an independent prognostic biomarker in EMPD.

## 2. Materials and Methods

### 2.1. Study Design and Patients

This retrospective cohort study included patients diagnosed with EMPD who were treated at our institution. This study included all patients with EMPD who were seen at the Department of Dermatology of our institution between April 2005 and December 2023. Sixty-three patients with available clinical data and formalin-fixed, paraffin-embedded tissue specimens were included. The diagnosis of EMPD was confirmed histopathologically in all cases. Patients with insufficient tissue samples or incomplete clinical follow-up data were excluded from the analysis.

Clinical information, including age at diagnosis, sex, lymph node status, and follow-up data, was obtained from medical records. Age was stratified using a cutoff value of 75 years. Lymph node swelling was assessed based on clinical examination and imaging studies at diagnosis. This study was conducted in accordance with the principles of the Declaration of Helsinki and was approved by the institutional review board of our institution. Due to the retrospective nature of the study, the requirement for obtaining written informed consent was waived, and an opt-out consent procedure was applied in accordance with institutional guidelines. Information regarding this study was disclosed on the institutional website, and patients were given the opportunity to opt out.

### 2.2. Histopathological Evaluation

Histopathological evaluation was performed using hematoxylin and eosin-stained sections. Dermal invasion was defined as the infiltration of Paget cells beyond the epidermis into the dermis and was assessed by experienced dermatopathologists who were blinded to the clinical outcomes. STING expression was evaluated using a binary classification (positive vs. negative) based on the presence or absence of distinct cytoplasmic staining in tumor cells as previously described [11]. Cases showing clear cytoplasmic staining in tumor cells were classified as STING-positive, whereas cases with absent or minimal staining were classified as STING-negative. This evaluation was independently performed by at least two investigators who were blinded to the clinical and survival data. Discrepancies between evaluators were resolved through a joint review to achieve consensus.

### 2.3. Immunohistochemical Analysis of STING Expression

Immunohistochemical staining was performed using a rabbit polyclonal anti-STING antibody (TMEM173; catalog no. 19851-1-AP, Proteintech, Rosemont, IL, USA). Paraffin-embedded tissue sections were deparaffinized and rehydrated through graded alcohols. Antigen retrieval was performed in citrate buffer (pH 9.0) for 15 min. Endogenous peroxidase activity was blocked by incubation with hydrogen peroxide for 5 min. After washing with distilled water, sections were incubated with the primary antibody diluted at 1:2000 for 60 min at room temperature.

After washing with distilled water and phosphate-buffered saline, sections were incubated with a secondary antibody for 30 min. Signal detection was performed using Histofine Simple Stain MAX-PO (R) (Nichirei Biosciences, Tokyo, Japan). Sections were developed with 3,3′-diaminobenzidine for 8 min, followed by rinsing in distilled water. Nuclei were counterstained with Mayer’s hematoxylin for 3 min and differentiated for 5 min. Finally, sections were dehydrated, cleared, and mounted for microscopic examination.

**Figure 1:**
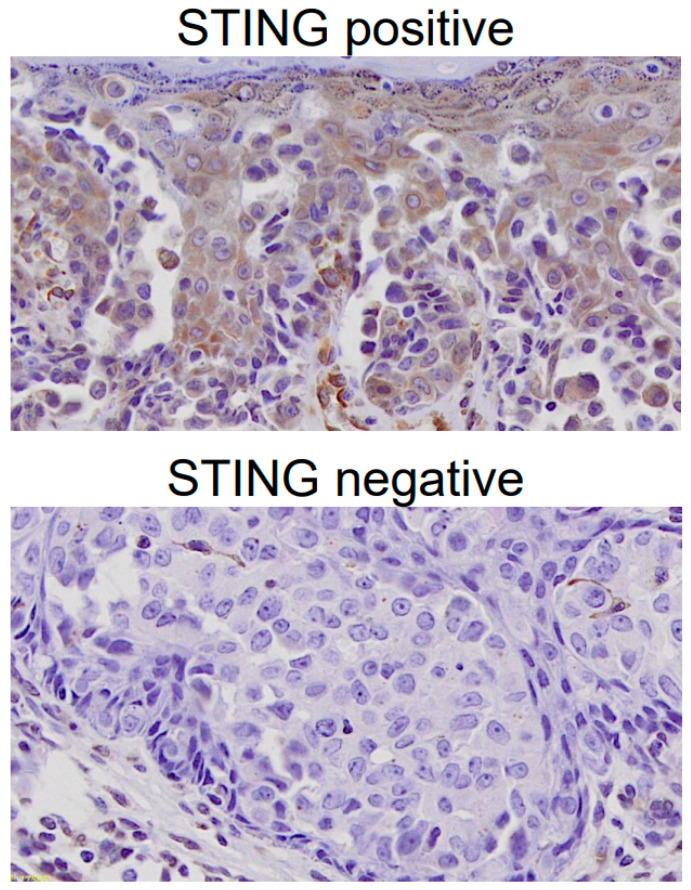
Representative immunohistochemical staining for STING in extramammary Paget’s disease tissue specimens. STING-positive tumors show distinct cytoplasmic staining in Paget cells, whereas STING-negative tumors exhibit absent or minimal cytoplasmic staining. STING, stimulator of interferon genes (×200).

STING expression was evaluated independently by at least two investigators who were blinded to the clinical and survival data. Cases were classified as STING-positive or STING-negative according to predefined criteria. Discrepancies between evaluators were resolved through a joint review to achieve consensus. Representative images of STING-positive and STING-negative tumors are shown in Figure 1.

### 2.4. Survival Analysis

Patient survival was calculated from the date of diagnosis to the date of death or last follow-up. Overall survival, defined as the duration from diagnosis to death from any cause or last follow-up, was used as the primary endpoint. Survival curves were estimated using the Kaplan–Meier method and compared between groups using the log-rank test.

### 2.5. Statistical Analysis

Associations between STING expression and clinicopathological variables were evaluated using appropriate statistical tests. Continuous variables were analyzed using non-parametric tests, and categorical variables were compared using Fisher’s exact test.

Cox proportional hazards regression analysis was performed to identify prognostic factors associated with patient survival. Variables that showed statistical significance in univariate analysis were included in a multivariate Cox regression model to adjust for potential confounding factors. Hazard ratios (HRs) and 95% confidence intervals (CIs) were calculated.

Kaplan–Meier analyses were performed using GraphPad Prism version 9.5.0 (GraphPad Software, San Diego, CA, USA). Univariate and multivariate Cox regression analyses were conducted using SPSS Statistics (version 27, IBM Corp., Armonk, NY, USA). All statistical tests were two-sided, and a *p* value of less than 0.05 was considered statistically significant.

## 3. Results

### 3.1. Patient Demographics and Baseline Clinicopathological Characteristics

Sixty-three patients with available clinical data and formalin-fixed, paraffin-embedded tissue specimens were included. The baseline clinical and pathological characteristics of the study population are summarized in Table 1. Patients were stratified by age using a cutoff value of 75 years; 38 patients (60%) were aged ≥75 years, whereas 25 patients (40%) were aged <75 years. The cohort consisted of 33 male (52%) and 30 female (48%) patients.

Lymph node swelling at diagnosis was observed in 16 patients (25%), while no lymph node involvement was evident in 47 patients (75%). Histopathological evaluation revealed dermal invasion in 31 patients (49%), whereas disease was confined to the epidermis in 32 patients (51%). Immunohistochemical analysis demonstrated positive STING expression in 26 patients (41%), and negative STING expression in 37 patients (59%).

### 3.2. Distribution of Clinicopathological Features According to STING Expression

To clarify the clinical significance of STING expression in EMPD, clinicopathological features were compared between STING-positive and STING-negative groups (Table 2). Age distribution did not differ significantly between the two groups. Among patients aged ≥75 years, 16 were STING-positive and 22 were STING-negative, while among patients aged <75 years, 10 were STING-positive and 15 were STING-negative (*p* > 0.9999).

Sex distribution was not significantly different between the two groups. Male patients tended to be more prevalent in the STING-negative group (23 vs. 10), whereas female patients tended to be more frequently observed in the STING-positive group (16 vs. 14); however, this difference did not reach statistical significance (*p* = 0.0775).

In contrast, marked differences were observed in tumor aggressiveness-related parameters. Lymph node swelling was detected in 12 of 37 patients with negative STING expression, compared with only in 4 of 26 patients with positive STING expression, indicating a significantly higher incidence of lymph node involvement in the STING-negative group (*p* = 0.0352). Similarly, dermal invasion was evident in 26 patients with negative STING expression, but only in 5 patients with positive STING expression. Conversely, dermal invasion was more frequently absent in the STING-positive group (21 patients) than in the STING-negative group (11 patients). This difference was highly significant (*p* < 0.0001).

### 3.3. Prognostic Impact of STING Expression on Patient Survival

**Figure 2:**
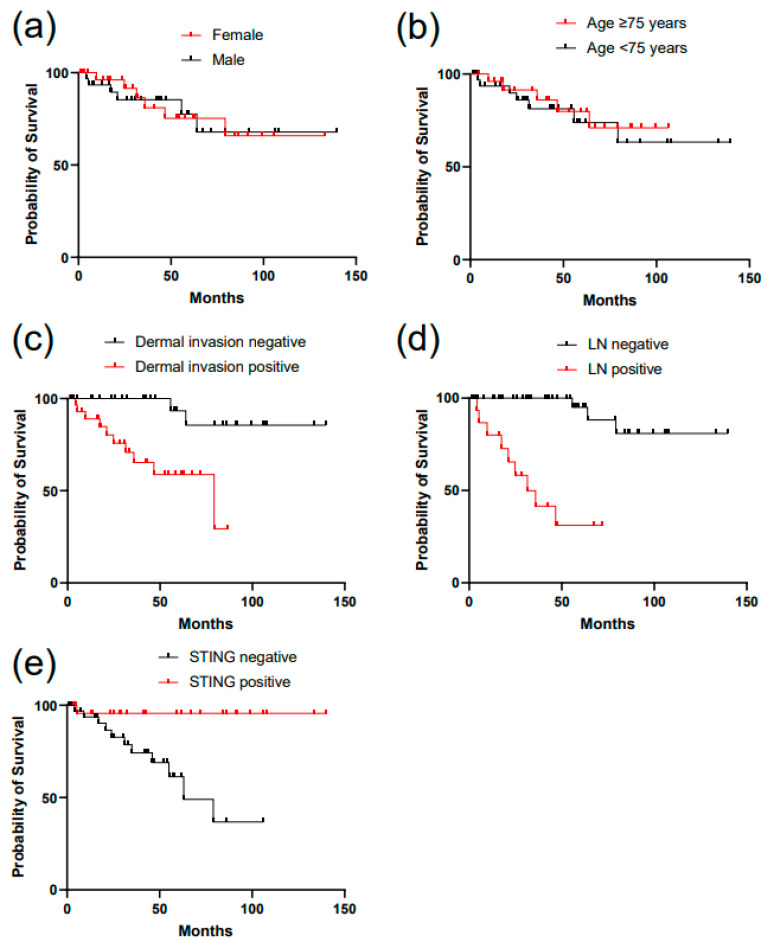
Survival analysis according to STING expression and clinicopathological parameters in extramammary Paget’s disease. Kaplan–Meier survival analyses of patients with extramammary Paget’s disease stratified according to (**a**) sex, (**b**) age at diagnosis (<75 vs. ≥75 years), (**c**) presence or absence of dermal invasion, (**d**) LN involvement at diagnosis, and (**e**) STING expression. Overall survival was compared between groups using the log-rank test. STING, stimulator of interferon genes; LN, lymph node.

To further elucidate the prognostic significance of STING expression in EMPD, survival outcomes were analyzed according to STING immunoreactivity. Kaplan–Meier analysis of overall survival revealed a significantly better prognosis in patients with positive STING expression than in those with negative STING expression (Figure 2).

Notably, the survival curves for the two groups began to diverge at an early stage of follow-up and remained clearly separated throughout the observation period, indicating that the impact of STING expression on patient prognosis was not transient but sustained over time. Patients with negative STING expression consistently exhibited a higher incidence of adverse events, whereas those with positive STING expression showed prolonged survival with fewer outcome events.

These findings suggest that STING expression reflects intrinsic differences in tumor behavior and disease progression. The consistent separation of the Kaplan–Meier curves for the two groups further supports the robustness of STING expression as a prognostic marker, rather than a surrogate for short-term disease severity.

### 3.4. Identification of Prognostic Factors by Univariate and Multivariate Analyses

To systematically identify clinicopathological factors associated with patient prognosis, univariate Cox proportional hazards regression analysis was first performed using clinically relevant variables, including age, sex, lymph node swelling, dermal invasion, and STING expression (Table 3).

In univariate analysis, lymph node swelling was identified as a strong adverse prognostic factor, with a markedly increased risk of poor outcome (HR 19.870, 95% CI 4.203–93.924, *p* < 0.001). Dermal invasion was also significantly associated with an unfavorable prognosis (HR 2.938, 95% CI 1.355–6.371, *p* = 0.006), indicating that deeper tumor infiltration correlated with an increased mortality risk. In contrast, STING expression demonstrated a significant protective effect, as patients with positive STING expression exhibited a substantially improved survival than patients with negative STING expression (HR 0.285, 95% CI 0.101–0.804, *p* = 0.018). Neither age nor sex showed a statistically significant association with patient outcomes in the univariate model.

To determine whether these variables exerted independent prognostic effects, factors that showed statistical significance in the univariate analysis were subsequently entered into a multivariate Cox proportional hazards regression model to adjust for potential confounding interactions among variables. The multivariate analysis showed that lymph node swelling remained a highly significant and independent predictor of poor prognosis (HR 30.184, 95% CI 3.288–277.065, *p* = 0.003), underscoring its dominant impact on survival in patients with EMPD.

Importantly, STING expression retained its prognostic significance after multivariate adjustment, emerging as an independent favorable prognostic factor (HR 0.196, 95% CI 0.048–0.801, *p* = 0.023). This finding indicates that the association between STING expression and improved survival was not attributable to confounding by lymph node status or tumor invasion depth. In contrast, dermal invasion did not show statistically significant association in the multivariate model (HR 1.555, 95% CI 0.629–3.846, *p* = 0.339), suggesting that its prognostic impact may be mediated, at least in part, through its association with lymph node involvement and STING expression.

## 4. Discussion

In this study, we demonstrated that loss of STING expression is strongly associated with aggressive clinicopathological features and poor prognosis in EMPD. Dermal invasion and lymph node swelling were significantly more common in STING-negative tumors, and overall survival was markedly worse in patients with negative STING expression. Importantly, multivariate Cox regression analysis identified STING expression as an independent favorable prognostic factor, even after adjustment for established predictors such as lymph node involvement and dermal invasion. These findings indicate that STING expression reflects underlying biological processes associated with tumor progression and patient outcomes, rather than merely serving as a surrogate marker of advanced disease. Importantly, this association remained significant in multivariate analysis after adjustment for established prognostic factors.

Recent studies have identified several clinicopathological prognostic factors in EMPD, including dermal invasion and lymph node involvement [12,13]. Our findings are consistent with these reports and further suggest that STING expression may provide additional prognostic information. Once invasion occurs, prognosis deteriorates sharply and therapeutic options become limited [14]. In this context, mechanisms governing immune surveillance and tumor–host interactions possibly play a pivotal role in determining whether tumors remain indolent or acquire invasive and metastatic potential. Our findings suggest that impairment of STING signaling may be involved in the transition toward a more aggressive phenotype, potentially allowing tumor cells to evade immune-mediated control.

The STING pathway is the central mediator of innate immune sensing and antitumor immunity through induction of type I interferon responses and activation of downstream adaptive immune mechanisms [15]. In many solid tumors, reduced STING activity has been implicated in immune evasion, diminished antitumor immune infiltration, and resistance to immunotherapy [16,17]. However, the biological and prognostic roles of STING signaling are highly tumor-type-dependent. The present findings pertaining to EMPD align more closely with the aggressive paradigm, suggesting that STING expression may reflect not only the immune microenvironment but also intrinsic tumor cell biology relevant to disease aggressiveness.

The strong inverse association observed between STING expression and both dermal invasion and lymph node swelling further supports the role of STING signaling in restraining invasive and metastatic behavior in EMPD. Dermal invasion represents a critical pathological milestone that precedes lymphatic spread, and lymph node involvement remains one of the most powerful predictors of survival in this disease. Although dermal invasion was significantly associated with prognosis in univariate analysis, it was not independently significant in the multivariate model, whereas STING expression was. This finding suggests that STING expression may reflect biological processes related to immune surveillance and tumor progression, including modulation of tumor–host interactions and antitumor immune responses, rather than implying a direct mechanistic role. A similar pattern has been reported in hematological malignancies such as lymphomas, in which reduced STING expression in tumor cells has been associated with an aggressive clinical behavior and a poor prognosis, independent of conventional pathological risk factors [11]. These observations support the concept that STING signaling functions upstream of overt pathological progression by integrating tumor-intrinsic and immune-related regulatory mechanisms.

Beyond its role in antitumor immune surveillance, STING signaling is also a regulator of tumor cell-intrinsic processes relevant to cancer progression, including DNA damage sensing, cellular stress responses, and control of cell survival and proliferation [18,19]. In this context, loss of STING expression may directly contribute to tumor growth and activation by permitting the survival and expansion of genetically unstable tumor cells [20]. In EMPD, tumor cells often persist within the epidermis for prolonged periods before acquiring invasive and metastatic abilities [14]. Impairment of STING signaling may facilitate this transition by attenuating DNA damage-induced signaling and apoptotic pathways, thereby enabling tumor cells to tolerate genomic instability and undergo clonal expansion with an enhanced invasive potential [19,20]. Such a mechanism is consistent with observations in other malignancies, including lymphomas, in which reduced STING expression is associated with aggressive tumor behavior independent of conventional pathological features. The strong association among STING expression loss, dermal invasion, and lymph node involvement observed in this study supports the concept that STING signaling acts as an upstream regulator of tumor progression rather than a passive marker of disease stage. Together, these findings suggest that STING expression may reflect upstream biological processes involved in tumor progression, rather than merely representing a passive marker of disease stage.

This study has several limitations. Its retrospective design and the relatively small sample size inherent to this rare disease may limit generalizability. In addition, STING expression was assessed by immunohistochemistry, which may be subject to methodological variability, including differences in antigen retrieval conditions, antibody performance, and interpretation of staining. Although these factors may influence the assessment of STING expression, the use of a binary classification approach and independent evaluation by multiple blinded observers likely reduces this variability, and the overall conclusions are unlikely to be substantially affected. Furthermore, functional analyses to directly elucidate the mechanistic role of STING signaling in EMPD progression were not performed. The relatively wide CIs observed in multivariate analyses may be a result of the limited cohort size. Therefore, validation in larger, independent cohorts and mechanistic studies exploring STING regulation and downstream signaling in EMPD are needed to confirm and extend our findings. In addition, STING expression was assessed using a binary classification rather than a fully quantitative cell-based scoring system, which may limit the granularity of the analysis. Furthermore, STING expression in adjacent non-lesional skin and healthy controls was not evaluated in this study, which limits the ability to determine baseline expression levels.

## 5. Conclusions

In conclusion, our study demonstrates that loss of STING expression is closely associated with aggressive clinicopathological features and independently predicts a poor prognosis in EMPD. These findings highlight STING expression as a promising prognostic biomarker and provide new insights into the immunobiology of EMPD, underscoring the importance of tumor-type-specific evaluation of innate immune signaling pathways.

## Figures and Tables

**Table 1:** The clinical characteristics of this study.

Variables	Number
Age	
≥75	38
<75	25
Sex	
Male	33
Female	30
Lymph node swelling	
Positive	16
Negative	47
Dermatol invasion	
Positive	31
Negative	32
STING	
Positive	26
Negative	37

**Table 2:** The different characteristics depending on STING expression.

Variables	STING Positive	STING Negative	*p*-Value
Age			>0.9999
≥75	16	22	
<75	10	15	
Sex			0.0775
Male	10	23	
Female	16	14	
Lymph node swelling			0.0352
Positive	4	12	
Negative	22	15	
Dermatol invasion			<0.0001
Positive	5	26	
Negative	21	11	

**Table 3:** The univariate and multivariate analysis.

	Univariate Analysis		Multivariate Analysis	
Variables	HR (Range)	*p*-Value	HR (Range)	*p*-Value
Age	1.130 (0.636–2.011)	0.676	1.191 (0.628–2.259)	0.593
Sex	1.016 (0.575–1.794)	0.956	1.170 (0.585–2.341)	0.657
Lymph node swelling	19.870 (4.203–93.924)	<0.001	30.184 (3.288–277.065)	0.003
Dermal invasion	2.938 (1.355–6.371)	0.006	1.555 (0.629–3.846)	0.339
STING	0.285 (0.101–0.804)	0.018	0.196 (0.048–0.801)	0.023

## Data Availability

The data supporting the findings of this study are available from the corresponding author upon reasonable request.
